# Autoregulation and Virulence Control by the Toxin-Antitoxin System SavRS in Staphylococcus aureus

**DOI:** 10.1128/IAI.00032-18

**Published:** 2018-04-23

**Authors:** Wen Wen, Banghui Liu, Lu Xue, Zhongliang Zhu, Liwen Niu, Baolin Sun

**Affiliations:** a CAS Key Laboratory of Innate Immunity and Chronic Disease, University of Science and Technology of China, Hefei, Anhui, China; b School of Life Sciences, University of Science and Technology of China, Hefei, Anhui, China; c Hefei National Research Center for Physical Sciences at the Microscale, Hefei, Anhui, China; New York University School of Medicine

**Keywords:** Staphylococcus aureus, toxin-antitoxin system, autoregulation, virulence control

## Abstract

Toxin-antitoxin (TA) systems play diverse physiological roles, such as plasmid maintenance, growth control, and persister cell formation, but their involvement in bacterial pathogenicity remains largely unknown. Here, we have identified a novel type II toxin-antitoxin system, SavRS, and revealed the molecular mechanisms of its autoregulation and virulence control in Staphylococcus aureus. Electrophoretic mobility shift assay and isothermal titration calorimetry data indicated that the antitoxin SavR acted as the primary repressor bound to its own promoter, while the toxin SavS formed a complex with SavR to enhance the ability to bind to the operator site. DNase I footprinting assay identified the SavRS-binding site containing a short and long palindrome in the promoter region. Further, mutation and DNase I footprinting assay demonstrated that the two palindromes were crucial for DNA binding and transcriptional repression. More interestingly, genetic deletion of the *savRS* system led to the increased hemolytic activity and pathogenicity in a mouse subcutaneous abscess model. We further identified two virulence genes, *hla* and *efb*, by real-time quantitative reverse transcription-PCR and demonstrated that SavR and SavRS could directly bind to their promoter regions to repress virulence gene expression.

## INTRODUCTION

Staphylococcus aureus is a ubiquitous human pathogen responsible for increasing numbers of infectious morbidity and mortality in both communal and hospital settings (1, 2). These infections range from superficial skin lesions to life-threatening inflammation, sepsis, endocarditis, and toxic shock (3). S. aureus exhibits its pathogenicity via numerous virulence factors, including toxins, adhesins, and secreted exoproteins (4). These virulence factors are cumulative and modulated by diverse regulatory factors, such as the *agr* quorum-sensing system and Sar family proteins, which give them a vast ability to survive, to induce infection, and to evade host immune defense (5–7). Therefore, virulence regulators have been proposed to be alternative targets for new therapeutics against staphylococcal infections.

Toxin-antitoxin (TA) systems were initially characterized as components of low-copy-number plasmids and later discovered on bacterial chromosomes. They have been found in diverse archaea and bacteria and play broad biological roles (8). TA systems typically consist of a stable toxin that can cause cell death by disrupting an essential cellular process and a labile antitoxin that can prevent the toxin from exerting its toxicity by forming a complex with toxin (9). It is currently thought that TA systems have a general role in stabilization of genomic parasites and growth control under stress stimuli (10, 11). Recent studies have demonstrated a great diversity among TA systems (12, 13). The type I TA system TisAB/IstR and the type II TA system HipBA were associated with persister cell formation (14). It was also found that the MazEF TA module is upstream of the *sigB* locus, and its promoter activity is essential for full *sigB* expression (15) and β-lactam susceptibility (16). MqsRA, as the most significant TA system with a regulatory role, was found to be involved in persister cell formation (17), biofilm formation (18, 19), quorum sensing (19), and cell motility (20).

Type II TA systems that consist of two proteins are the largest and best-studied TA system classes. In a typical type II TA system, the antitoxin can form a stable protein-protein complex with the toxin to counteract its toxicity. Under stress conditions, the antitoxin is degraded by cellular proteases, freeing the toxin to inhibit growth by inhibiting translation or replication (21, 22). The majority of type II toxins are nonspecific endoribonucleases (RNases) degrading total cellular RNA (23), but some toxins, like MazF (24) and MqsR (19, 25), are sequence-specific RNases degrading mRNA with substrate specificity. These toxins can be viewed as global regulators that regulate gene expression at the posttranscriptional level by differential mRNA decay (26). TA-driven RNA interference has been implicated in the regulation of antibiotic resistance and virulence in numerous bacteria, including Escherichia coli (26), Mycobacterium tuberculosis (27), S. aureus (28, 29), Clostridium difficile (30), and enterococci (31). Interestingly, researchers have found that not only do toxins modulate cell phenotypes by degrading mRNA but some antitoxins also affect cell physiology by regulating other cellular systems (32). The antitoxin MqsA regulates more than its own synthesis, as it is the first antitoxin that has been shown to bind directly to the *mqsRA*, *spy*, *mcbR*, *cspD*, and *rpoS* promoters and regulate the transcription of these loci (17, 20, 25, 32). This creates a new paradigm wherein antitoxins of TA systems can be viewed as regulators. The occurrence of this mechanism is a very attractive concept of global regulation by TA systems and awaits thorough experimental verification. Although TA systems play a variety of physiological roles in plasmid maintenance, growth control, and persister cell formation, their involvement and function in bacterial pathogenicity remain elusive.

In this study, we characterized a novel type II TA system, SavRS, in S. aureus. We demonstrated that antitoxin SavR and toxin-antitoxin complex SavRS can bind to the specific motif of its own promoter for autoregulation. More importantly, we found that SavR and SavRS can negatively regulate the expression of virulence genes *hla* and *efb* by directly binding to their promoters. The virulence regulation was further investigated through hemolytic activity and a mouse subcutaneous abscess model.

## RESULTS

### SavRS is a type II TA system in S. aureus.

**FIG 1:**
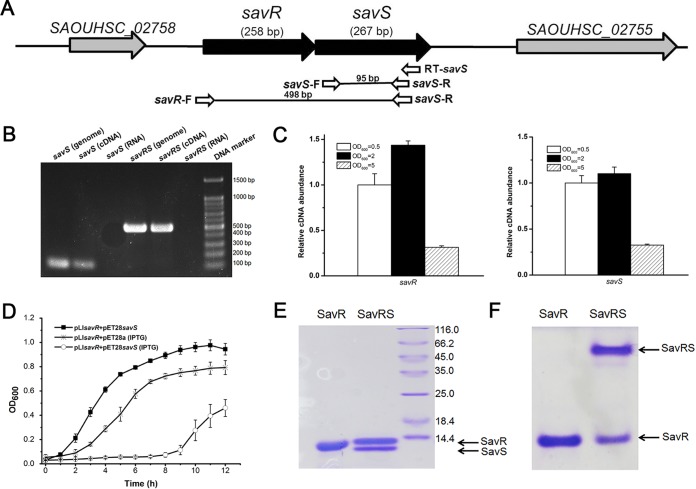
SavRS is a type II toxin-antitoxin system. (A) Genetic organization of the *savRS* operon and the location of primers used in the PCR assay. (B) *savS* and *savR* are cotranscribed. Gel electrophoresis analysis of PCR products amplified with genomic DNA, reverse-transcribed cDNA with specific primer RT-*savS*, and RNA. (C) The expression of *savR* and *savS* is growth phase dependent. (D) Inhibition of cell growth by SavS. The strain harboring plasmids pET28*savS* and pLI*savR* was induced with 0.5 mM IPTG, and the growth curve was monitored for 12 h. (E) Protein electrophoresis by SDS-PAGE. (F) SavS and SavR form a stable complex in native PAGE.

We identified a putative type II toxin-antitoxin system, SavRS, in S. aureus NCTC8325 that shares homologous sequence with YefM-YoeB in E. coli (33), as determined by the TAfinder tool (34). The putative toxin and antitoxin were encoded by *SAOUHSC_02756* and *SAOUHSC_02757* and contained a 1-bp overlap sequence. The antitoxin gene was designated *savR* (*Staphylococcus aureus* autoregulation and virulence regulator) in accordance with its function in S. aureus, and the toxin gene was named *savS* since it is located downstream of *savR* (Fig. 1A). The upstream gene *savR* encodes a protein (85 residues) with 40.7% consensus and 24.4% identity to YefM in E. coli, as aligned by Vector NTI (Thermo Fisher Scientific). The downstream gene *savS* encodes a protein (88 residues) with 31.5% consensus and 21.3% identity to YoeB. To verify whether or not *savR* and *savS* are cotranscribed, reverse transcriptase PCR of *savS* was performed with RNA extracted from S. aureus NCTC8325 by specific primer RT-*savS*. Using primer pairs spanning the open reading frames (ORFs) of *savR* and *savS* and compared to the results with the same amount of genomic DNA or RNA only, positive signals were detected (Fig. 1B). We further performed 3′ rapid amplification of cDNA ends (RACE) analysis to determine the sequences of *savR* mRNA. Sequence analysis indicated that the *savR* mRNA contains the ORFs of both *savR* and *savS*. These data indicated that *savR* and *savS* were cotranscribed from the *savR* promoter.

We further detected *savRS* transcription in different growth phases. As shown in Fig. 1C, the transcript levels of *savR* and *savS* are higher in early exponential and mid-logarithmic phases, indicating that their expression is growth phase dependent. To verify the predicted function of these genes, the expression plasmids pET22*savS* and pET28*savR* were constructed in E. coli TOP10. Transformation of the plasmid pET22*savS* with a tightly regulated T7-dependent promoter into the expression strain E. coli BL21 could not yield colonies on agar plates, suggesting that even the low background expression of SavS is lethal to bacterial cells. When the plasmids pET22*savS* (Amp^r^) and pET28*savR* (Kan^r^) were cotransformed into E. coli BL21, cells displayed normal growth, suggesting that the expression of SavR could counteract the toxicity of SavS. To characterize the toxicity of SavS, the plasmid pET28*savS* (Kan^r^), which expresses toxin with an isopropyl-β-d-thiogalactopyranoside (IPTG)-induced promoter, and the plasmid pLI*savR* (Amp^r^), which expresses antitoxin continuously, were cotransformed into E. coli BL21. The cells harboring two plasmids exhibited cell growth arrest under IPTG induction, while cells without induction exhibited normal growth (Fig. 1D). Together, the toxicity of SavS is lethal to bacterial cells, and this toxicity can be reversed by the expression of SavR.

To further characterize the *savRS* system, we expressed and purified the SavR and SavRS complex (Fig. 1E) in E. coli as described in Materials and Methods. The toxin SavS (without hexahistidine tag) interacted with the hexahistidine-tagged antitoxin SavR to form a complex. From native PAGE analysis, the SavRS complex, appearing with a higher-molecular-weight band than that of SavR alone, was observed at the top of the gel (Fig. 1F), indicating that SavR and SavS could form a stable complex by protein-protein interaction. These data suggest that SavRS is a type II toxin-antitoxin system in S. aureus.

### SavR and SavRS repressed the transcription of *savRS*.

**FIG 2:**
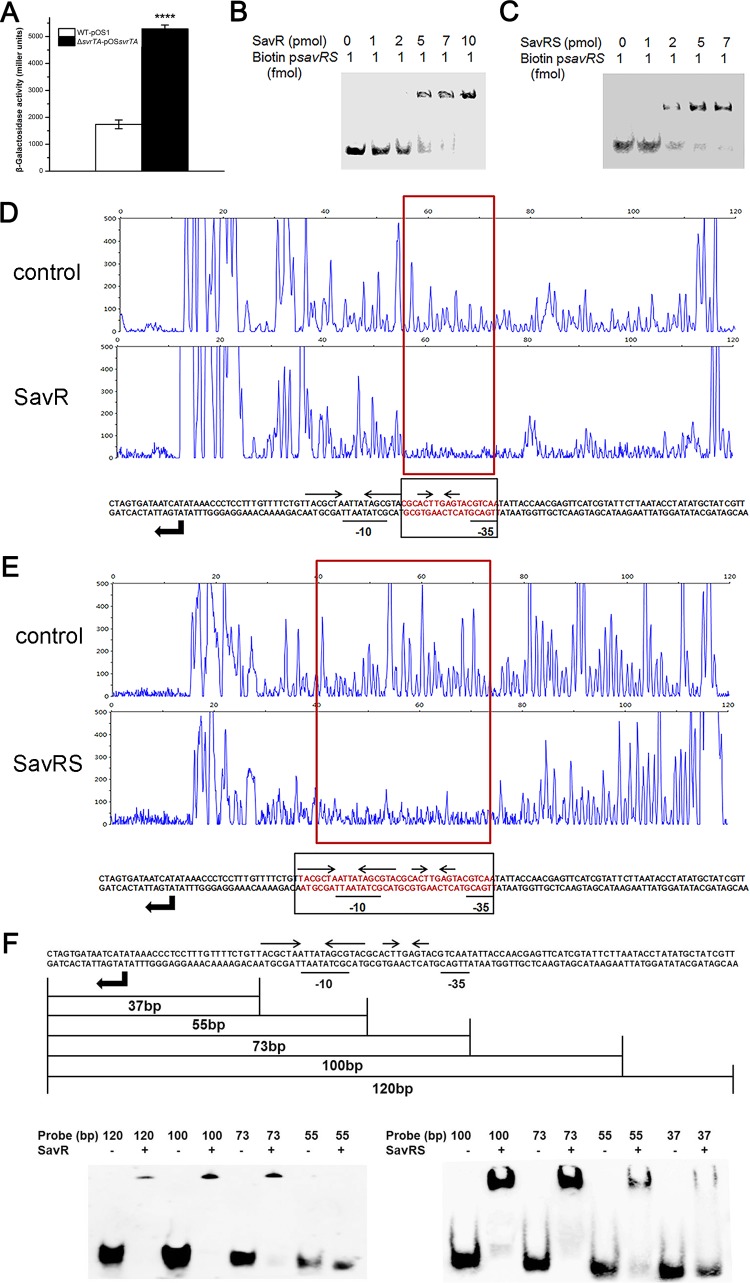
SavR and SavRS repress the expression of *savRS*. (A) The β-galactosidase activity driven by the *savRS* promoter in the WT and the *savRS* mutant strains. ****, *P* < 0.0001. (B) SavR bound to the promoter region of *savRS*. (C) SavRS bound to the promoter region of *savRS*. (D) The 18-bp recognition site for SavR in its own promoter region was identified by DNase I footprinting. (E) The 36-bp binding site for SavRS in the promoter region was identified by DNase I footprinting. The regions protected from DNase I digestion are marked by red boxes. The binding sequences are marked by red letters and black boxes. The locations of the long and short palindromes are marked by inverted arrows. (F) Determination of binding ability of SavR and SavRS with truncated probes by EMSA.

In most type II TA systems, the antitoxin and toxin proteins play an important role in transcriptional autoregulation under stress conditions (35, 36). To investigate transcriptional regulation, we tried to construct the *savR* mutant and *savRS* mutant strains. Unfortunately, we failed to generate a *savR* mutant, presumably due to toxicity of SavS in the absence of the antitoxin. We then constructed the *lacZ* fusion reporter plasmid pOS*savRS* with its own promoter region and detected the β-galactosidase activities of the wild-type (WT) strain and the *savRS* mutant strain. As shown in Fig. 2A, the absence of *savRS* significantly increased *savRS* promoter activity, suggesting that SavRS can repress the transcription of the *savRS* operon.

To determine whether the transcription of *savRS* is under the direct control of SavRS, electrophoretic mobility shift assay (EMSA) was performed with the 267-bp biotin-labeled putative promoter region and proteins. EMSA data indicated that SavR and SavRS could retard the mobility of the *savRS* promoter in a dose-dependent manner (Fig. 2B and C), revealing that the antitoxin SavR, serving as a repressor, autoregulates the transcription of the *savRS* operon by directly binding to its promoter region while the toxin SavS acts as a corepressor.

The binding sites for SavR and SavRS in the *savRS* promoter region were further identified by DNase I footprinting. SavR specifically protected the 18-bp region of the *savRS* promoter between the −35 and −10 transcriptional boxes from DNase I digestion (Fig. 2D). SavRS specifically protected the 36-bp region of the *savRS* promoter covering the SavR-protected region (Fig. 2E). The EMSA results (Fig. 2F) are consistent with the DNase I footprinting data, demonstrating that the *savRS* promoter region is bound more avidly by the SavRS complex than by SavR alone.

### The DNA palindromes played a crucial role in DNA binding and transcription repression.

**FIG 3:**
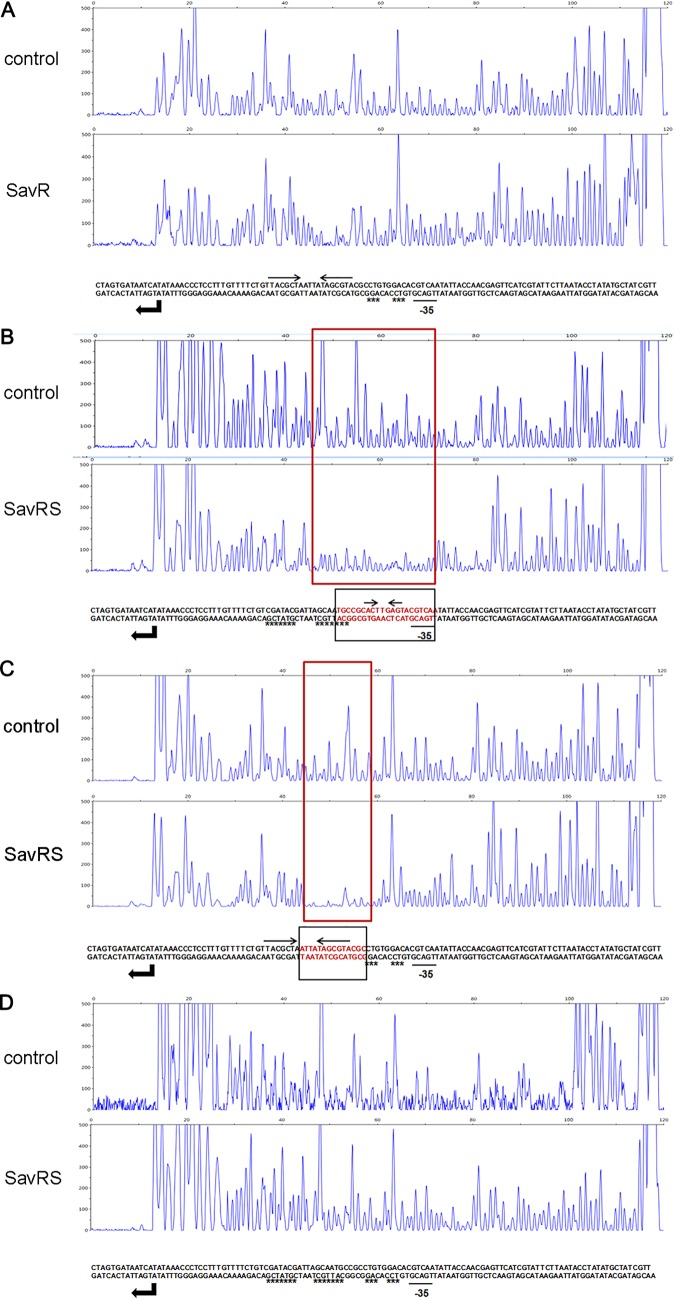
DNA palindromes are crucial to DNA binding. (A) DNase I footprinting assay of SavR with the mutated short palindrome. (B) DNase I footprinting assay of SavRS with the mutated long palindrome. (C) DNase I footprinting assay of SavRS with the mutated short palindrome. (D) DNase I footprinting assay of SavRS with the mutated long and short palindromes. The signal peaks were detected by short tandem repeat sequencing. The red boxes denote the regions protected from DNase I digestion by SavR (A) and SavRS (B, C, and D) compared with the sequencing results without protein. The relative dispositions of sequences are illustrated in the lower panel. The locations of the long and short palindromes are marked by inverted arrows. The substitution mutation sites are marked by stars.

The primary region of the *savRS* operon protected by SavR against DNase I attack includes a short palindrome sequence (5′-ACT**AGT-3′) (Fig. 2D). The primary region protected by SavRS includes a long palindrome sequence (5′-TACGCTA****TAGCGTA-3′) and a short palindrome sequence (5′-ACT**AGT-3′) (Fig. 2E). An asterisk represents any base in these palindrome sequences. We hypothesized that the palindrome sequence plays a crucial role in DNA binding and transcription repression. To test this hypothesis, multiple substitution mutations were introduced into the long palindrome, the short palindrome, and both the long and short palindromes in the operator site. As we expected, SavR failed to protect the operator site harboring mutations in the short palindrome from DNase I digestion (Fig. 3A). For the SavRS complex, the ability to protect the operator site was weakened when the long palindrome or the short palindrome was mutated (Fig. 3B and C). This protection was abolished when both palindromes were disrupted (Fig. 3D). The disruption of palindromes dramatically impaired the interaction of SavR and SavRS with their own promoters. These results emphasize the critical role of palindromes in DNA binding and transcription repression.

### SavS enhanced SavR binding to the operator site of the promoter sequence.

**FIG 4:**
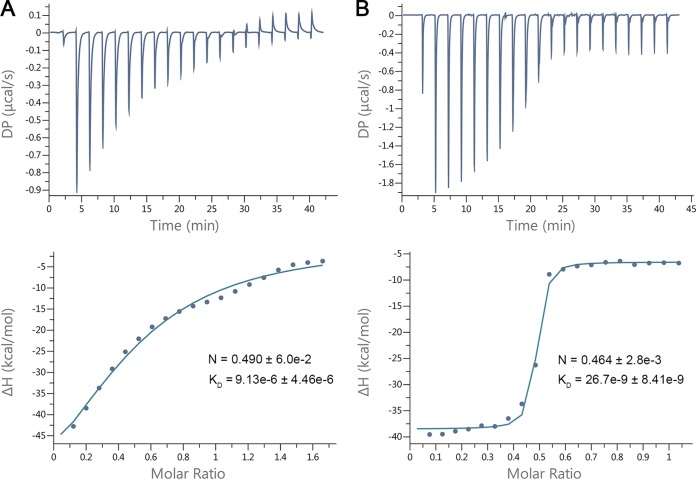
SavS enhances SavR binding to the operator site. (A) The ITC fitting result of antitoxin SvrR with DNA. The thermodynamic data were collected from injections of DNA into SavR, and the binding isotherm was fitted to a one-site binding model after subtraction of blank titration heats. (B) The ITC fitting result of toxin-antitoxin complex SvrRS with DNA. The data were processed using the method described for panel A. DP is a measured power differential between the reference and sample cells to maintain a zero temperature between the cells. Δ*H* represents enthalpy.

SavR bound to the short palindrome in the promoter region, while SavRS recognized both the short and long palindromes to regulate the transcription of the *savRS* operon. To quantify the interaction of DNA with proteins, isothermal titration calorimetry (ITC) was employed to measure the thermodynamic parameters. The 36-bp sequence containing the whole binding site was synthesized and titrated into SavR and SavRS, respectively. We first measured the binding affinity (*K_D_*) of the 36-bp sequence to SavR. The *K_D_* value was fit to 9.13 ± 4.46 μmol/liter, with an overall stoichiometry of ≈1:2 (Fig. 4A). We then measured the binding affinity of the 36-bp sequence to SavRS. The ITC result showed that the binding capacity of the nucleotides to SavRS is ∼300-fold stronger than that of SavR, and the *K_D_* value was fit to 26.7 ± 8.41 nmol/liter with an overall stoichiometry of ≈1:2 (Fig. 4B). The thermodynamic data determined by ITC are listed in Table 1. Taken together, these results indicate that SavS can enhance the interaction between SavR and the operator site of the promoter sequence.

**TABLE 1:** Thermodynamic data determined by isothermal titration calorimetry^*a*^

Sample	*N*	*K_D_* (M)	Δ*H* (kcal/mol)	Δ*G* (kcal/mol)	−*T*Δ*S* (kcal/mol)
SavR	0.490 ± 6.0e−2	9.13e−6 ± 4.46e−6	−80 ± 26.2	−6.88	73.1
SavRS	0.464 ± 2.8e−3	26.7e−9 ± 8.41e−9	−31.9 ± 0.539	−10.3	21.6

a *N*, stoichiometry of binding; *K_D_*, binding affinity; Δ*H*, enthalpy; Δ*G*, Gibbs free energy; −*T*Δ*S*, entropy change.

### The *savRS* mutant strain exhibited increased hemolytic activity.

**FIG 5:**
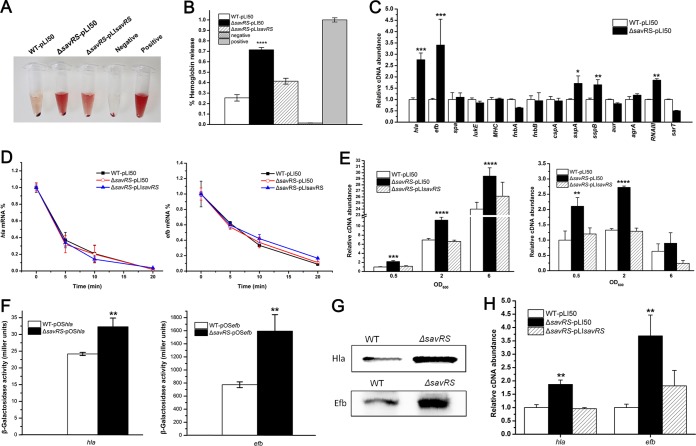
SavRS represses the expression of virulence genes *hla* and *efb*. (A) The *savRS* mutant strain exhibited increased hemolytic activity. The hemolytic activity assay was performed in 3% sheep erythrocytes. (B) The hemolytic activity was determined by absorption of supernatants at 543 nm. The percentage of hemolytic activity was calculated relative to the positive control, which was regarded as 100% hemolytic activity. ****, *P* < 0.0001. (C) The transcript levels of some virulence genes in the WT and the *savRS* mutant strains by qRT-PCR assay. *, *P* < 0.05; **, *P* < 0.01; ***, *P* < 0.001. (D) Analysis of the mRNA half-life in the WT, the *savRS* mutant, and the *savRS* complemented strains. (E) The growth-phase-dependent transcription of *hla* and *hlb* in the WT and the *savRS* mutant strains. **, *P* < 0.01; ***, *P* < 0.001; ****, *P* < 0.0001. (F) The β-galactosidase activity under the control of the *hla* promoter and *efb* promoter in WT and *savRS* mutant strains. **, *P* < 0.01. (G) Comparison of the protein expression levels of WT and *savRS* mutant strains by Western blotting. (H) Analysis of mRNA levels in hemolytic activity assay. The strains were incubated with 3% sheep erythrocytes for 20 min, the cells were collected, and the mRNA levels were analyzed by qRT-PCR. **, *P* < 0.01.

In previous studies, some type II TA systems could bind to more than their own promoters to modulate quorum sensing, biofilm formation, and dispersal (20). To explore their additional impact on cell physiology, a hemolytic activity assay was performed with the WT strain containing pLI50 (WT-PLI50), the *savRS* mutant strain containing pLI50 (Δ*savRS*-pLI50), and the *savRS* mutant strain containing pLI*savRS* (Δ*savRS*-pLI*savRS*). The absorption was measured at 543 nm, and the percentage of hemolytic activity was calculated. The *savRS* mutant strain exhibited significantly increased hemolytic activity compared to that of the WT strain (Fig. 5A and B). This alteration could be restored by a complementary plasmid containing *savRS*, suggesting that SavRS can regulate virulence gene expression in S. aureus.

### SavRS is a negative regulator of virulence genes.

To determine whether the expression of virulence genes was altered in the *savRS* mutant, real-time quantitative reverse transcription-PCR (qRT-PCR) was performed to examine the mRNA levels of some virulence genes (Fig. 5C). The transcript level of *hla*, encoding alpha-hemolysin (37), was increased ∼2.8-fold in the *savRS* mutant strain compared with that of the WT strain. Meanwhile, the transcript level of *efb*, encoding extracellular fibrinogen-binding protein (38), was increased ∼3.4-fold. To verify that the increase of hemolytic activity is predominantly due to the expression changes of virulence genes, we determined the mRNA half-lives of *hla* and *efb*. As shown in Fig. 5D, the mRNA levels in the *savRS* mutant strain were reduced at similar rates in the WT and complementary strains. Moreover, to characterize the virulence control by SavRS, we detected the transcript levels of *hla* and *efb* in a growth phase-dependent manner (Fig. 5E). The transcript levels of *hla* and *efb* displayed similar increases in the *savRS* mutant strain compared with those of the WT strains and the complementary strains in all growth phases. The β-galactosidase activity detected by *lacZ* fusion reporter plasmids pOS*hla* and pOS*efb* gave consistent results (Fig. 5F). Western blot assay indicated that the expression levels of Hla and Efb were also significantly increased in the *savRS* mutant strain (Fig. 5G). These data demonstrated that SavRS can repress the expression of *hla* and *efb*. To detect the direct linkage of *hla* and *efb* expression to hemolytic activity, we collected the bacterial cells in a hemolytic assay process, extracted total RNA, and performed qRT-PCR. The transcript levels of *hla* and *efb* were increased significantly in the *savRS* mutant strains after incubation with sheep erythrocytes (Fig. 5H). These data indicated that the increase of hemolytic activity is mainly caused by the changes of the expression levels of virulence genes.

### SavRS directly binds to the *hla* and *efb* promoters.

**FIG 6:**
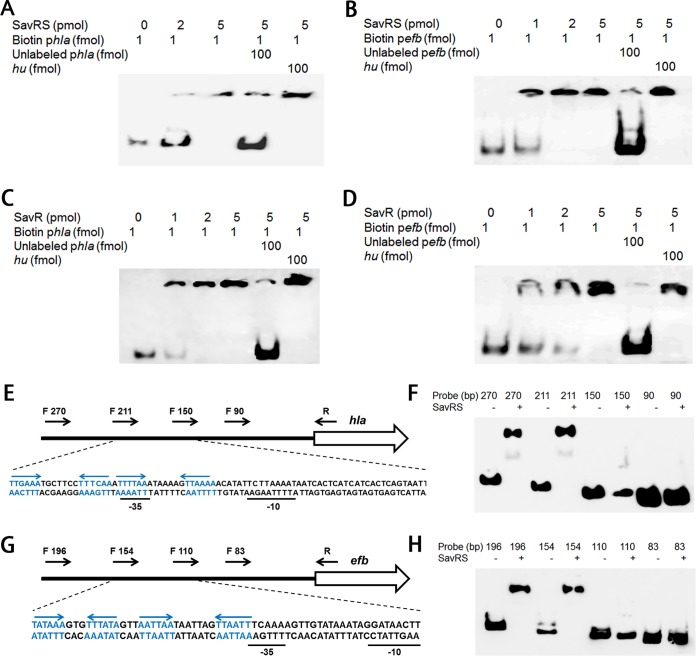
SavRS binds directly to the *hla* and *efb* promoters. (A) The toxin-antitoxin complex SavRS bound to the promoter region of *hla*. The unlabeled *hla* promoter probe served as the specific competitor, and the unlabeled *hu* ORF region served as the nonspecific competitor. (B) SavRS bound to the promoter region of *efb*. (C) SavR bound to the promoter region of *hla*. (D) SavR bound to the promoter region of *efb*. (E) The locations of *hla* truncated probes with different lengths are marked by black arrows. The partial relative dispositions of sequence are illustrated in the lower panel. The locations of the palindromes are marked by blue and inverted arrows. (F) EMSA analysis of SavRS with *hla* truncated probes. (G) The locations of *efb* truncated probes with different lengths. (H) EMSA analysis of SavRS with *efb* truncated probes.

To test whether *hla* and *efb* are under the direct control of SavRS, EMSA was performed with SavRS and biotin-labeled promoter sequences. As shown in Fig. 6A and B, SavRS could retard the mobility of the *hla* and *efb* promoter regions in a dose-dependent manner. This shifted band disappeared in the presence of an approximately 50-fold excess of the unlabeled target promoter DNA but not in the presence of a 50-fold excess of the unlabeled DNA fragment of *hu*. EMSA was also performed using the antitoxin SavR, and similar band shift patterns were observed with the *hla* and *efb* promoter regions (Fig. 6C and D). EMSA was also performed using RNA III promoter and SavR/SavrS and negative signals were detected, suggesting that SavR/SavRS cannot regulate RNA III by directly binding to its promoter. These data suggested that SavRS can bind to *hla* and *efb* promoters to regulate their expression to affect virulence.

To identify the SavRS recognition sequence in the promoters of virulence genes, truncated probes with different lengths were designed to perform EMSA (Fig. 6E and G). As shown in Fig. 6F and H, the ability of SavRS to bind to *hla* was abolished when the probe length was truncated from 211 bp to 150 bp (Fig. 6F). The ability of SavRS to bind to *efb* was abolished when the probe length was truncated from 154 bp to 110 bp (Fig. 6H). The recognition sequences of *hla* and *efb* both contain two adjacent palindromes. Since we first demonstrated that the palindromes in the *savRS* promoter region are crucial for DNA binding, it is reasonable to hypothesize that the palindromes in the promoter regions of virulence genes also play an important role in DNA binding and regulation. Interestingly, no conserved sequence has been found in these palindromes.

### Virulence assessment in subcutaneous abscess infection of mice.

**FIG 7:**
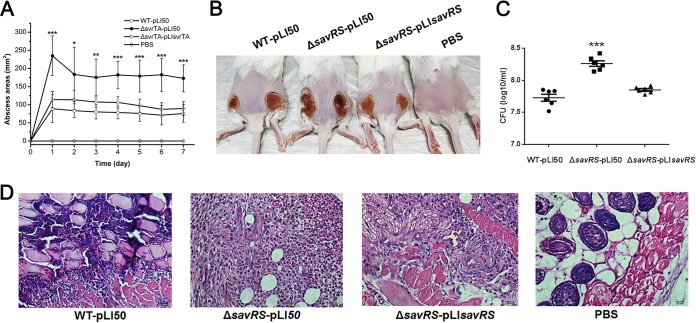
*savRS* mutant strain displays enhanced virulence in the mouse abscess infection model. (A) Mice were injected subcutaneously with 5 × 10^7^ cells. Abscess areas were measured daily. *, *P* < 0.05; **, *P* < 0.01; ***, *P* < 0.001. (B) Representative abscesses at 7 days after infection. (C) The abscesses were harvested at 7 days, and the number of CFU recovered from each abscess was determined. ***, *P* < 0.001. (D) Representative histological results (H&E stain).

To investigate the pathogenicity of the *savRS* mutant, the mouse subcutaneous abscess model was used to assess the contribution of SavRS to skin and soft-tissue infection. Mice were subcutaneously injected in both flanks with 5 × 10^7^ S. aureus cells by CFU counting, and the course of infection was monitored daily (Fig. 7A). The sizes of abscesses caused by the *savRS* mutant strain were significantly larger than those caused by the WT strain, which was further demonstrated in photographs of the skin lesions (Fig. 7B). In parallel with its increased lesion response, the bacterial colonization of the lesion skin in Δ*savRS*-infected mice was 4-fold higher than that in WT-infected mice after 7 days of infection. These alterations could be restored by the complementary strain (Fig. 7C). Histological examinations of the *savRS* mutant showed more extensive inflammation with leukocyte infiltration, destruction of the skin structure, and inflammation (Fig. 7D). Taking these findings together, SavRS can negatively regulate the expression of virulence genes to affect the pathogenicity of S. aureus.

## DISCUSSION

TA systems are commonly found in bacteria, and their physiological roles, such as cell growth arrest and persister cell formation, have been widely recognized. A number of studies suggest a regulatory role of TA systems that affect cell physiology, including biofilm formation, quorum sensing, cell motility, and antibiotic resistance. The majority of their regulatory roles are implemented by toxins containing site-specific endoribonuclease activity, and the antitoxin MqsA can regulate more than its own synthesis through its DNA-binding motif (20, 32). In bacterial pathogens, discovery of the virulence control and elucidation of the regulatory mechanism seem to be significant and interesting.

At least four different families of TA systems are predicted to exist in the opportunistic pathogen S. aureus, but their physiological roles are elusive (39). In one recent study, the authors found that knockout of three TA systems in S. aureus had no effect on persister cell formation in exponentially growing or stationary-phase cells with ciprofloxacin, oxacillin, vancomycin, and rifampin (40). It has been reported that an S. aureus MazEF deletion mutant was more susceptible to β-lactam antibiotics (16), but the molecular mechanism was not provided. The toxin pemK_Sa_ (toxin-antitoxin system PemIK_Sa_), located on the plasmid pCH91, is a sequence-specific endoribonuclease recognizing the tetrad sequence U↓AUU and is involved in global regulation of staphylococcal virulence by altering translation of large pools of genes (28).

Here, we have characterized an S. aureus type II TA system with novel features, SavRS. SavRS can perform autoregulation and virulence control by directly binding to its own promoter and the promoter regions of virulence genes. The toxin SavS was lethal to cells, and the antitoxin SavR could form a stable complex with SavS to counteract its toxicity. A long palindrome sequence containing a 5′-TACGCTA****TAGCGTA-3′ motif and a short palindrome sequence containing a 5′-ACT**AGT-3′ motif between the −35 and −10 transcriptional boxes were identified in the *savRS* promoter region. The specific binding site was further identified by DNase I footprinting, and the data indicate that SavR, as a transcriptional repressor, can only bind to the short palindrome. By forming a complex with SavS as a corepressor, SavRS can bind to both the long and the short palindromes. Introduction of substitution mutations in the palindromes and DNase I footprinting analysis indicated that the palindromes are crucial for DNA binding and autoregulation.

It has been recognized that TA systems are involved in multiple regulatory pathways and physiological processes in bacteria (13, 26). In our study, we were extremely interested in virulence regulation by TA systems. We performed hemolytic activity assays in the WT and the *savRS* mutant strains and observed increased hemolytic activity in the *savRS* mutant strain. Further qRT-PCR assay identified the virulence genes *hla* and *efb*, the former of which encodes an alpha-hemolysin toxin associating into hexamers or heptamers to form a pore in the membrane (37), and the latter encodes an extracellular fibrinogen-binding protein that prevents platelet aggregation and is involved in the pathogenesis of wound infections (41). Our data further demonstrate that SavR and SavRS could bind directly to the promoters of *hla* and *efb* to regulate the expression of these genes. The virulence regulation was assessed in a mouse subcutaneous abscess model, further illustrating the regulatory role of SavRS in virulence expression *in vivo*.

S. aureus can cause a great diversity of infections, which often involve the coordination of multiple virulence genes and regulatory factors. Several of these virulence regulators, such as the *agr* system, Sar family proteins, and RNA III, have been well characterized in S. aureus, while the involvement of TA systems in virulence regulation in pathogenic bacteria remains largely unknown. Our study demonstrates that a novel type II toxin-antitoxin system, SavRS, can act as a virulence regulator to repress the expression of some virulence genes. Our findings may not only provide novel insight into the virulence regulation network but also broaden the diverse physiological roles of TA systems in S. aureus and other pathogenic bacteria.

## MATERIALS AND METHODS

### Bacterial strains, plasmids, and growth conditions.

The bacterial strains and plasmids used in this study are listed in Table 2. E. coli strains were cultured with shaking (220 rpm) in lysogeny broth (LB) medium or on lysogeny broth agar (LA) at 37°C. S. aureus and its derivative strains were cultured with shaking (220 rpm) in tryptic soy broth (TSB) medium or on tryptic soy agar (TSA) at 37°C. Media were supplemented with appropriate antibiotics (E. coli, 100 μg/ml ampicillin and 50 μg/ml kanamycin; S. aureus, 15 μg/ml chloromycetin) to maintain plasmids where necessary.

**TABLE 2:** Strains and plasmids used in this study

Strain or plasmid	Description	Source or reference
Strains		
Escherichia coli		
Trans T1	Cloning strain	TransGen
BL21(DE3)	Expression strain	TransGen
Staphylococcus aureus		
NCTC8325	WT	NARSA
RN4220	8325-4, r^−^	NARSA
WT-pLI50	NCTC8325, WT with plasmid pLI50	This study
Δ*savRS*	NCTC8325, *savRS*-deleted strain	This study
Δ*savRS*-pLI50	*savRS*-deleted strain with plasmid pLI50	This study
Δ*savRS*-pLI*savRS*	Complementary strain of Δ*savRS*, Δ*savRS* with pLI*savRS*	This study
Plasmids		
pBTs	Shuttle vector, temp sensitive, Amp^r^ Cm^r^	42
pBT*savRS*	pBTs derivative, for *savRS* deletion, Amp^r^ Cm^r^	This study
pBT*efb-his*	pBTs derivative, for His tag insertion in *efb*	This study
pET22b(+)	Expression vector with a hexahistidine tag, Amp^r^	Novagen
pET28a(+)	Expression vector with a hexahistidine tag, Kan^r^	Novagen
pET22*savS*	Protein SavS expression vector, Amp^r^	This study
pET28*savR*	Protein SavR expression vector with a hexahistidine tag, Kan^r^	This study
pET28*savS*	Protein SavS expression vector with a hexahistidine tag, Kan^r^	This study
pLI50	Shuttle cloning vector, Amp^r^ Cm^r^	Addgene
pLI*savRS*	pLI50 with *savRS* and its promoter, Amp^r^ Cm^r^	This study
pOS1	Shuttle vector, with *lacZ* ORF lacking first 6 amino acids, Amp^r^ Cm^r^	43
pOS*savRS*	POS1 derivative, harboring 267-bp region of *savRS* promoter and 18 bp of *savR* coding sequence from strain NCTC8325, Amp^r^ Cm^r^	This study
pOS*hla*	POS1 derivative, harboring 300-bp region of *hla* promoter and 18 bp of *hla* coding sequence from strain NCTC8325, Amp^r^ Cm^r^	This study
pOS*efb*	POS1 derivative, harboring 299-bp region of *efb* promoter and 18 bp of *efb* coding sequence from strain NCTC8325, Amp^r^ Cm^r^	This study

### Construction of the *savRS* mutant strain.

The oligonucleotide primers used in this study are listed in Table 3. To construct the *savRS* deletion strain, the upstream and downstream fragments of *savRS* were amplified from S. aureus NCTC8325 genomic DNA using the *savRS*-up-F/*savRS*-up-R and *savRS*-down-F/*savRS*-down-R set of primers and ligated by overlap to form an up-down fragment. The product fragment was digested with KpnI/SalI and then ligated into the temperature-sensitive shuttle vector pBTs. The resulting plasmid pBT*savRS* was constructed using E. coli transT1 and transformed into S. aureus RN4220 by electroporation for modification and then transformed into S. aureus NCTC8325. The mutant strains that had allelic replacement were screened as anhydrotetracycline-resistant and chloramphenicol-sensitive colonies and were further confirmed by PCR and sequencing.

**TABLE 3:** Oligonucleotide primers used in this study

Primer	Oligonucleotide (5′–3′)	Application
*savRS*-up-F	GTGCAGCGGAATTCGAGCTCGGTACCGCGATTATCAAATGCTTTCG	*savRS* deletion
*savRS*-up-R	TGAATATTCAATATCTGAAT	*savRS* deletion
*savRS*-down-F	ATTCAGATATTGAATATTCAATAAACCCTCCTTTGTTTTC	*savRS* deletion
*savRS*-down-R	TAAAGCTTGCATGCCTGCAGGTCGACCTGTGCTTTAATTGCTTTTC	*savRS* deletion
*savRS*-c-F	CCGGAATTCGTCGCTATTTTCCCTACACT	Complementation
*savRS*-c-R	CGGGGTACCTGTGAACGATAGCATATAGG	Complementation
*savS*-protein-F	GGAATTCCATATGAGCAATTACACGGTTAAGATTAAA	Protein expression
*savS*-protein-R	CCGCTCGAGTTAATCATAATGTGACCATGCCGATAG	Protein expression
*savR*-protein-F	GGAATTCCATATGATTATCACTAGCCCTACAGAAGCG	Protein expression
*savR*-protein-R	CCGCTCGAGTTAAAGATTATCCCAATCAATATCATCT	Protein expression
P*savRS*-F	GAGTTTTTTTAAGCGGATTC	EMSA
P*savRS*-R	CTAGTGATAATCATATAAACCCTCC	EMSA
P*savRS*-biotin-R	CTAGTGATAATCATATAAACCCTCC	EMSA (5′ biotin)
P*hla*-F	TTCAACTTTGACTAACCCTC	EMSA
P*hla*-R	TTTCATCATCCTTCTATTTT	EMSA
P*hla*-biotin-R	TTTCATCATCCTTCTATTTT	EMSA (5′ biotin)
P*efb*-F	ATACCTTCAGACACCAACAT	EMSA
P*efb*-R	AATACCTATTGCCGCTAATG	EMSA
P*efb*-biotin-R	AATACCTATTGCCGCTAATG	EMSA (5′ biotin)
P*savRS*-F120	AACGATAGCATATAGGTATT	DNase I footprinting
P*savRS*-FAM-R	CTAGTGATAATCATATAAACCCTCC	DNase I footprinting
UP-L mutant-R	GCAATGCCGCACTTGAGTACGTCAATA	Palindrome mutation
D-L mutant-F	GTACTCAAGTGCGGCATTGCTAATCGTATCGACAGAAAACAAAGGAGGGTT	Palindrome mutation
UP-S mutant-R	TAGCGTACGCCTGTGGACACGTCAATATTACCAACGAG	Palindrome mutation
D-S mutant-F	GTGTCCACAGGCGTACGCTATAATTAGCG	Palindrome mutation
UP-LS mutant-R	GCAATGCCGCCTGTGGACACGTCAATATTACCAACGAG	Palindrome mutation
D-LS mutant-F	GTGTCCACAGGCGGCATTGCTAATCGTATCGACAGAAAACAAAGGAGGGTT	Palindrome mutation
*hla*-RT-F	AAAGTAGGCTGGAAAGTGAT	qRT-PCR
*hla*-RT-R	TAGCGAAGTCTGGTGAAA	qRT-PCR
*efb*-RT-F	GCGGCAATAGGTATTACTAC	qRT-PCR
*efb*-RT-R	ACCATCATTGTACTCTACGA	qRT-PCR
RT-*savS*	TTAATCATAATGTGACCATG	Reverse transcription
*savS*-R	GACCATGCCGATAGTATT	PCR
*savS*-F	TTAGAGCGATATTCAAGAAGA	PCR
*savR*-F	GCCCTACAGAAGCGAGAA	PCR
P*savRS*-lacZ-F	CCGGAATTCGAGTTTTTTTAAGCGGATTC	pOS*savRS*
P*savRS*-lacZ-R	CGCGGATCCGGAGGGCTAGTGATAATCAT	pOS*savRS*
P*hla*-lacZ-F	CCGGAATTCTTCAACTTTGACTAACCCTC	pOS*hla*
P*hla*-lacZ-R	CGCGGATCCGGGACTATACGTGTTTTCATTT	pOS*hla*
P*efb*-lacZ-F	CCGGAATTCATACCTTCAGACACCAACAT	pOS*efb*
P*efb*-lacZ-R	CGCGGATCCGGTATCAATTTATTTTTCATGT	pOS*efb*
*efb*-up-F	CGGGGTACCTCTATGAGATTATACCTTCAGACAC	His tag insertion
*efb*-up-R (his)	GTGATGATGATGATGATGTTTAACTAATCCTTGTTTTAATAC	His tag insertion
*efb*-down-F (his)	CATCATCATCATCATCACTAAAACTTCAATCGTTGCTGTTATC	His tag insertion
*efb*-down-R	ACGCGTCGACGCACTAGAACTGGAAATGATAA	His tag insertion

### Complementation of the *savRS* deletion strain.

A 712-bp fragment encompassing the open reading frame of *savRS* and its native promoter was amplified from S. aureus NCTC8325 genomic DNA. The fragment was digested with EcoRI/KpnI and cloned into the shuttle plasmid pLI50 to derive the plasmid pLI*savRS*. The recombinant plasmid was transformed by electroporation into S. aureus RN4220 for modification and subsequently into S. aureus NCTC8325 to derive the complemented strain C*savRS*. The NCTC8325 WT strain and the *savRS* mutant strain (Δ*savRS*) were also transformed with the empty plasmid pLI50 as the control strains, resulting in the WT-pLI50 and Δ*savRS*-pLI50 strains, respectively.

### Expression and purification of SavR and SavRS proteins.

To express the SavR protein, the protein expression plasmid pET28*savR* was transformed into E. coli BL21(DE3) cells, and the transformant was cultivated in LB at 37°C to an optical density at 600 nm (OD_600_) of 0.6 and induced with 0.5 mM IPTG at 16°C for an additional 20 h. The cells were harvested and lysed by sonication in lysis buffer (20 mM Tris-HCl, pH 8.0, 100 mM NaCl). The SavR protein was purified by three steps of chromatography (histidine affinity by Ni^2+^-nitrilotriacetic acid, size selection by Superdex G75, and anion-exchange chromatography by HiTrap). To obtain the SavRS protein complex, the plasmids pET28*savR* and pET22*savS* were cotransformed into E. coli BL21(DE3). Ampicillin and kanamycin were used to maintain plasmids. The protein SavS (without hexahistidine tag) interacts with the hexahistidine-tagged SavR protein to form the complex. The complex was also purified by three steps of chromatography as described above.

### Total RNA isolation, cDNA generation, and qRT-PCR.

Overnight cultures of S. aureus were diluted 1:100 in TSB and grown at 37°C. The cells were collected at the indicated cell density and processed with 1 ml RNAiso (TaKaRa) in combination with 0.1-mm-diameter zirconia-silica beads in a FastPrep-24 automated system (MP Biomedicals). The residual DNA was removed with RNase-free DNase I (TaKaRa). Transcription analysis of *savRS* was performed by reverse transcription-PCR using Moloney murine leukemia virus reverse transcriptase (TaKaRa) with primer RT-*savS*, and the PCR products were analyzed with the primers *savS*-F/*savS*-R and *savS*-F/*savR*-R. Reverse transcription for qRT-PCR analysis was performed using a PrimeScript first-strand cDNA synthesis kit (TaKaRa) with random primers. Real-time PCR was carried out with SYBR premix *Ex Taq* (TaKaRa) using a StepOne real-time PCR system (Applied Biosystems). The quantity of cDNA measured was normalized to the *hu* cDNA abundance. The primers used in this study are listed in Table 3.

### RACE analysis.

The 3′ ends of *savR* were determined by rapid amplification of cDNA ends (RACE) using the 3′-full RACE core set kit (ver.2.0; TaKaRa). The PCR products were cloned into T vector (TransGen) for sequencing.

### Electrophoretic mobility shift assay.

The biotin-labeled DNA fragments containing the promoter region were amplified from the S. aureus genomic DNA. The biotin-labeled DNA fragment was incubated at 25°C for 20 min with purified protein in 10 μl of incubation buffer (20 mM Tris-HCl, pH 8.0, 100 mM NaCl). Samples were run on a 3.5% native polyacrylamide gel in 1× Tris-borate-EDTA buffer at 100 V for 20 min. DNA was transferred to a nylon membrane at 400 mA for 30 min and then UV cross-linked. Chemiluminescence was performed with a chemiluminescent nucleic acid detection module (Pierce) according to the manufacturer's instructions. The images were obtained using an ImageQuant LAS 4000 mini (GE, Piscataway, NJ, USA). The unlabeled fragments of each promoter were added to the labeled fragments at a ratio of approximately 50 to 1 as specific competitors. The unlabeled fragment of the *hu* ORF region (50-fold) was added as a nonspecific competitor.

### DNase I footprinting assay.

The 120-bp PCR fragment was generated with the primers P*savRS*-F120 and P*savRS*-FAM-R, the latter of which was labeled with 6-carboxylfluorescein (6-FAM) in the 5′ terminus. The reactions were carried out with 1 pmol 5′ 6-FAM-labeled template DNA and various amounts of the purified SavRS protein at 25°C for 30 min in reaction buffer (20 mM Tris-HCl, pH 8.0, 100 mM NaCl, 5 mM MgCl_2_, 1 mM CaCl_2_). The reaction mixture was supplied with 0.3 U/100 μl of DNase I (Promega) and incubated for 30 s at 37°C. The DNA fragments were extracted with phenol-chloroform, precipitated with 95% ethanol, washed with 75% ethanol, dried, resuspended in double-distilled water (ddH_2_O), and then detected by short tandem repeat (STR) sequencing. The protected region of SavRS was derived by comparing the sequencing results to those without SavRS by using Peak Scanner software v1.0 (Applied Biosystems).

### ITC.

The ITC experiment was carried out on a MicroCal PEAQ ITC calorimeter (GE Healthcare) at 25°C. The 36-bp DNA fragment was synthesized by Sangon Biotech, dissolved with buffer (20 mM Tris-HCl, pH 8.0, 100 mM NaCl), and diluted to 200 μM. The protein concentrations of SavR and SavRS were determined by both the bicinchoninic acid protein assay kit and NanoDrop (Thermo) and then diluted to appropriate concentrations. Heats of dilutions were measured in blank titrations by injecting the DNA into buffer, and the dilution heats were subtracted from the binding heats. Curve fitting to a one-site binding model was performed with the ITC data analysis module provided with the MicroCal PEAQ ITC calorimeter.

### Construction of LacZ reporter plasmids.

To construct the reporter plasmid pOS*savRS* for detection of *savRS* expression, the 267-bp fragment of the *savRS* native promoter and a region of the first 18 bp of the *savRS* coding sequence were amplified from S. aureus NCTC8325 genomic DNA with the primer P*savRS*-lacZ-F/R. The fragment was digested with BamHI/EcoRI and cloned into the shuttle vector pOS1 to generate reporter plasmid pOS*savRS*. The same protocol was followed to construct the reporter plasmids pOS*hla* and pOS*efb* using primers P*hla*-lacZ-F/R and P*efb*-lacZ-F/R, respectively. Constructed plasmids were transformed into E. coli Trans T1 for amplification and S. aureus RN4220 for modification and were subsequently transformed into S. aureus NCTC8325.

### β-Galactosidase activity assay.

For β-galactosidase activity assays, stationary-phase cultures of the WT strain and the *savRS* mutant strain containing different LacZ reporter plasmids were diluted 1:100 into TSB with chloromycetin. Cells were collected at the early log phase (OD_600_ of 1.0) and lysed for 30 min at 37°C by the use of 100 μl ABT LSA buffer (60 mM K_2_HPO_4_, 40 mM KH_2_PO_4_, 100 mM NaCl, 0.01% Triton X-100, 50 μg/ml lysostaphin). We next added 100 μl ABT buffer and 100 μl 4 mg/ml ONPG (*o*-nitrophenyl-β-d-galactopyranoside) to initiate the reaction. The samples were incubated at 37°C until a yellow color became apparent, and then 1 ml Na_2_CO_3_ was added to stop the reaction. The galactosidase activity analysis was calculated according to the method described previously.

### Hemolytic activity assay.

The WT strain with plasmid pLI50, the *savRS* mutant strain with plasmid pLI50, and the *savRS* complementary strain were cultured in TSB at 37°C and collected until the postexponential phase (OD_600_ of 2.5). Bacterial culture supernatants (100 μl) were mixed with 900 μl phosphate-buffered saline (PBS) buffer containing 3% sheep erythrocytes, and the mixtures were incubated at 37°C for 20 min. The absorption of supernatant at 543 nm was measured after centrifugation. A mixture with 1,000 μl ddH_2_O containing 3% sheep erythrocytes was used as the positive control, and a mixture with 1,000 μl PBS containing 3% sheep erythrocytes was used as the negative control. The percentage of hemolytic activity was calculated relative to the positive control, which was regarded as 100% hemolytic activity.

### mRNA half-life assay.

Overnight cultures of S. aureus were inoculated at 1:100 into TSB medium and grown for 4 h. Cultures were treated with rifampin (200 μg/ml) for 0, 5, 10, or 20 min. Cells then were collected after rifampin treatment and processed for RNA isolation, and then the *hla* and *efb* mRNA levels were measured by qRT-PCR.

### Western blot analysis.

To detect the expression level of Hla, bacterial cells at the stationary phase (OD_600_ of 5) were collected, lysed by lysostaphin at 37°C for 30 min, and then heated at 95°C for 10 min. The samples were separated by 12% SDS-PAGE and electrotransferred onto a polyvinylidene difluoride membrane (GE, Piscataway, NJ). The protein was detected with a rabbit anti-alpha-toxin antibody (Sigma) followed by horseradish peroxidase-conjugated sheep anti-rabbit antibodies (Pierce). To detect the expression level of Efb, a His_6_ tag in the 3′-terminal coding region of *efb* was inserted into the genome and anti-His tag antibody (GenScript) was used.

### Mouse subcutaneous abscess model.

Outbred, immunocompetent female BALB/c mice between 5 and 6 weeks of age were purchased from Beijing Vital River Laboratory Animal Technology Company. The hair on the back was removed by an animal shaver. Overnight cultures of S. aureus isolates in TSB were collected, washed twice, and diluted in sterile PBS. Viable cells were counted via CFU counting on TSB agar plates in order to quantify the infectious dose. Mice were inoculated with 5 × 10^7^ live S. aureus cells or PBS alone in both flanks of the back by subcutaneous injection. Abscess areas, assessed as the maximal length times width of the developing ulcers, were measured daily. The skin lesions were excised and homogenized in water after 7 days. The number of CFU recovered from each individual lesion was counted by serial dilution and plated onto TSB agar plates. For histopathological analyses, the skin lesions were placed in 10% formalin. Paraffin embedding and hematoxylin and eosin (H&E) staining were performed by Anhui Provincial Hospital.

### Statistics.

Statistical analysis was performed using Origin 8.5 and GraphPad Prism 5. Data were analyzed using unpaired *t* tests to compare two different conditions and analysis of variance for more conditions. All error bars show the standard errors of the means (SEM). All experiments were performed in biological triplicates.

All animal experiments were performed by following the guidelines adopted by the Ministry of Health of the People's Republic of China in June 2004. The protocol was approved by the Institutional Animal Care and Use Committee of the University of Science and Technology of China (USTCACUC1701005). All efforts were made to minimize suffering and the number of mice used.
